# Detecting reconciliation discrepancies in tabular data using transformers

**DOI:** 10.3389/frai.2026.1840051

**Published:** 2026-08-24

**Authors:** Carl Du Plessis, Mike Wa Nkongolo

**Affiliations:** 1 Department of Computer Science, University of Pretoria, Pretoria, South Africa; 2 Department of Informatics, University of Pretoria, Pretoria, South Africa

**Keywords:** data lakes, discrepancy detection in tabular datasets, entity matching, semantic embeddings, transformer models

## Abstract

**Introduction:**

Large organisations maintain heterogeneous datasets in data lakes, where schema variability, inconsistent formats, and semantic ambiguity complicate reconciliation. Achieving a unified view of entities requires methods that capture both structural equality and semantic relationships.

**Methods:**

We propose a transformer-based methodology that adapts pre-trained language models (PLMs) to tabular data by generating metadata-enriched embeddings (table title, column name, type, and statistics). These embeddings are compared using mutual top-*K* similarity, value-level verification, and Facebook AI Similarity Search (FAISS) for discrepancy detection. Ground-truth labels were established through manual annotation of 1,000 column pairs per dataset, with three annotators achieving substantial agreement (Cohen’s *κ* = 0.82).

**Results:**

Experiments on large-scale tabular data (185,909 tables) demonstrate that semantic embeddings efficiently uncover relationships and discrepancies, achieving precision of 0.958 at *τ* = 0.9 and F1-scores ranging from 0.77–0.87. Benchmarking against baselines (exact matching, Jaccard similarity, edit distance, TF-IDF/BM25, sentence-transformer embeddings, DeepJoin-style embeddings, and schema-name-only matching) shows that lexical baselines achieve high precision but poor recall, while semantic baselines capture relationships but underperform on heterogeneous tables. In contrast, our metadata-enriched transformer embeddings consistently achieved the highest F1-scores.

**Discussion:**

Unlike prior schema-aligned or query-driven approaches such as DeepJoin, WarpGate, and Lotus, this study introduces a unified reconciliation pipeline that integrates equality-based and semantic matching for large-scale tabular data. The key contribution is a scalable and generalisable reconciliation methodology that operationalises transformer architectures for automated discrepancy detection in heterogeneous environments, establishing both methodological novelty and practical effectiveness.

## 1 Introduction

In modern data-driven environments, organisations frequently need to reconcile data across heterogeneous systems, where the same real-world entities are represented using different schemas, formats, and conventions. This task, commonly referred to as data reconciliation or entity matching, is critical for ensuring data consistency, integrity, and reliability in downstream analytics and decision-making processes. Traditional approaches, including rule-based and statistical matching techniques (Samoilă, 2015), rely heavily on predefined schemas and handcrafted features, limiting their ability to generalise to previously unseen data sources. Recent advances in deep learning, particularly transformer-based architectures, have demonstrated remarkable success in capturing contextual and semantic relationships in unstructured data (Tabinda Kokab et al., 2022; Nassiri and Akhloufi, 2023; Rahali and Akhloufi, 2023). Despite these advances, their application to structured tabular data remains relatively underexplored, especially in the context of data reconciliation across heterogeneous datasets (van Breugel and van der Schaar, 2024). Tabular data presents unique challenges, including schema variability, inconsistent representations, and the need to capture both exact and semantic correspondences across columns and records. This study addresses these challenges by investigating the effectiveness of transformer-based models for relationship discovery and discrepancy detection in heterogeneous tabular data. Specifically, the research evaluates whether pre-trained language models (PLMs) can be adapted to identify both equality-based and semantic relationships across previously unseen datasets. Building on this, the study proposes a unified methodology that integrates relationship discovery with query-driven validation, enabling a scalable and generalisable approach to data reconciliation. By combining these components, the work advances the application of transformer architectures beyond unstructured text, positioning them as a viable solution for complex data integration tasks (Levene and Loizou, 2003; Harby and Zulkernine, 2022; Hai et al., 2023). To ensure focus and feasibility, the scope of this research is restricted to structured and semi-structured data sources, including datasets that can be represented in tabular form (e.g., JSON and XML). Unstructured data modalities such as images, audio, and video are excluded. The study focuses on column-level relationship discovery, considering both equality and semantic correspondences (Saïs et al., 2009), while excluding complex multi-table dependencies that require extensive domain-specific knowledge or business rule interpretation. The methodology leverages existing PLMs, with an emphasis on adaptation and optimisation for tabular data reconciliation rather than modification of the underlying transformer architecture (Mohammad et al., 2018). Experimental evaluation is conducted within a controlled lakehouse environment using AWS. The validation is performed on two large-scale datasets comprising approximately 30,000 and 1 million tables, reflecting realistic data integration scenarios. While the proposed approach is designed for generalisability across domains, the experiments focus on common business datasets, acknowledging that highly specialised domains may require further domain-specific adaptation.

### 1.1 Contributions

This study makes four primary contributions. First, it systematically evaluates transformer-based approaches for discovering both equality-based and semantic relationships in heterogeneous tabular datasets. Second, it proposes a unified framework that integrates relationship discovery with discrepancy detection, enabling end-to-end data reconciliation. Third, it demonstrates scalability and practical applicability within large-scale lakehouse environments. Finally, it provides empirical insights into the strengths and limitations of transformer-based models in tabular data reconciliation, laying the groundwork for future research on automated, intelligent data integration and validation systems.

### 1.2 Novelty of the approach

Unlike prior reconciliation methods such as DeepJoin, WarpGate, and Lotus, our framework introduces two methodological innovations:

1. Metadata-enriched embeddings: Each column is contextualised not only by its values but also by table-level metadata (title, column name, inferred type, and statistical descriptors). This enrichment improves semantic precision in heterogeneous datasets, whereas DeepJoin assumes schema alignment and WarpGate embeds columns independently without metadata.
2. Unified reconciliation pipeline: Our method integrates equality-based and semantic matching within a single transformer-embedding framework. Relationships are discovered via mutual top-*K* similarity, verified at the value level, and discrepancies detected using Facebook AI Similarity Search (FAISS)-based approximate nearest neighbour (ANN) search. Lotus focuses on query expansion with large language models (LLMs), but does not operationalise reconciliation at scale. In contrast, our pipeline is designed for large-scale, automated reconciliation across previously unseen tables.

Together, these innovations establish a generalisable methodology that moves beyond component reuse, demonstrating novelty in both embedding design and system integration. Specifically, we introduce transformation keys that integrate table title, column name, type, and statistical features into embeddings, and combine equality-based and semantic matching within a single framework. While components such as pre-trained embeddings, cosine similarity, and FAISS indexing are reused, the novelty lies in generalising reconciliation at scale with metadata-driven embeddings and mutual top-*K* verification. The manuscript is structured as follows: Section 2 reviews transformer-based embedding models for encoding tabular data, highlighting the novelty of this study. Section 3 describes the experimental datasets, design, environment, and preprocessing pipeline. Section 4 presents the performance of the proposed model in identifying and validating relationships, detecting reconciliation discrepancies, and analysing time complexity. Finally, Section 5 concludes the study.

## 2 Literature review

This literature review addresses the foundational challenges of automated reconciliation discrepancy detection. In their survey, Singh and Bedathur (2023) listed a number of transformer based embedding models for the encoding of tabular data (Supplementary Table S1).

Embedding models for tabular data should work out-of-the-box without extensive training. However, most tabular-specific models such as TabNet (Arik and Pfister, 2021) and SAINT (Somepalli et al., 2022) require training. Pre-trained models like TabPTM (Ye et al., 2025) generate row-level embeddings, which are less effective for relationship discovery where column-level representations are more suitable (Deng et al., 2022). Consequently, we focus on pre-trained embedding models that either natively support tabular data or can be adapted with minimal preprocessing, such as serialising columns or rows into text. When integrating heterogeneous data sources into platforms like data lakes, reconciliation challenges arise when entities may or may not represent the same real-world object (Saïs et al., 2009). Traditional similarity-based methods rely on schema attributes and handcrafted rules, limiting scalability (Roychowdhury et al., 2024; Gunnu et al., 2026). Deep learning approaches improve blocking and matching (Thirumuruganathan et al., 2021), but often assume identical schemas. Methods such as DeepJoin (Dong et al., 2023) and related work (Dong et al., 2021) encode column values as vectors to identify equality or semantic relationships (Glass et al., 2021), though they struggle with non-textual data and large tables due to transformer token limits (Herzig et al., 2020). WarpGate (Cong et al., 2022) extends this idea by embedding columns into high-dimensional spaces to capture semantic joinability, while Lotus (Patel et al., 2024) leverages LLMs for large-scale semantic queries (Iida et al., 2021). Other studies explore statistical correlation (He et al., 2015) or set similarity (Zhu et al., 2019), but these approaches remain limited to specific relationship types (Koloski et al., 2025). Collectively, these works establish the foundation for automated reconciliation discrepancy detection (Yin et al., 2020; Yu et al., 2021). Our study builds on this by targeting large, unseen datasets and developing a generalisable embedding-based reconciliation method that avoids reliance on handcrafted rules or rigid table structures. While prior methods such as DeepJoin, WarpGate, and Lotus focus primarily on schema-level or query-driven semantic joins, our approach introduces a unified reconciliation pipeline that integrates equality-based and semantic matching within a single transformer-embedding framework. Unlike DeepJoin, which assumes schema alignment, our method performs column-level relationship discovery across previously unseen tables using metadata-enriched embeddings. WarpGate embeds columns independently, whereas our approach contextualises each column with table-level and statistical metadata, improving semantic precision. Lotus leverages LLMs for query expansion, while our system operationalises reconciliation at scale through mutual top-K verification and FAISS-based discrepancy detection. This integration yields a generalisable, end-to-end methodology for large-scale tabular reconciliation.

## 3 Experimental design

The dataset consists of over one million GitHub CSV files sourced from the GitTables corpus (Hulsebos et al., 2023), representing relational tables accompanied by structural and semantic metadata. After consolidating duplicate table definitions within repositories, a total of 185,909 unique tables were retained. GitTables provides column-level semantic annotations from DBpedia (835 types) and Schema.org (677 types), summarised in Supplementary Table S2. Column-level semantic similarity was computed from text-based embeddings, enabling large-scale pairwise comparison across all columns (Supplementary Figure S1).

The lakehouse architecture was deployed on Amazon Web Services (AWS): raw Comma-Separated Values (CSV) files were converted into Parquet via AWS Glue and stored in Amazon S3 using the Apache Iceberg table format. Resulting column embeddings were stored in Amazon S3 Vectors to support scalable semantic retrieval (Venkatesan et al., 2026a).

In addition to large-scale reconciliation experiments, we conducted a controlled evaluation on a single representative table. The full table was treated as the reference, and derivative tables were created by randomly truncating 10, 25, 50, and 75% of the rows. Controlled truncation on a representative table demonstrated non-linear degradation of cosine similarity as rows were progressively removed. To generalise this finding, we extended the experiment to multiple representative tables across different domains. Results are summarised in Supplementary Table S3.

These results confirm that transformer-based embeddings are resilient to moderate data loss (25%) but degrade sharply when 50% of rows are removed, a pattern consistent across heterogeneous domains. Cosine similarity was then computed between the reference and each truncated version to quantify the effect of data removal on embedding accuracy (Supplementary Figure S2). This setup isolates the impact of missing records, allowing us to assess how robustness of the similarity measure decreases as the proportion of removed data increases. Ground-truth labels for column relationships and discrepancies were created through manual annotation of sampled subsets from the GitTables corpus.

### 3.1 Annotation methodology

To establish ground-truth labels, 1,000 column pairs per dataset were manually annotated by three data-science experts. Annotation guidelines defined semantic equivalence, partial overlap, and non-equivalence based on column metadata and value distributions. Disagreements were resolved through majority voting. Inter-annotator agreement was measured using Cohen’s Kappa (*κ* = 0.82), indicating substantial consistency. Although the full dataset exceeds one million tables, the annotated subset provides statistically representative coverage for evaluation. These annotations served as the reference for computing precision, recall, and F1-scores.

#### 3.1.1 Reproducibility

All experiments were executed in AWS eu-west-1 using Glue crawlers, Athena (service limit of 150 concurrent queries), and ECS Fargate tasks configured with 8,192 CPU units and 32,768 MiB memory. Random sampling and truncation were performed with fixed seeds to ensure reproducibility. FAISS IVFPQ was configured with nlist = 1,024 and nprobe = 16. Baselines (exact match, Jaccard, edit distance, TF-IDF/BM25, sentence-transformers, DeepJoin-style embeddings) were implemented using scikit-learn v1.3 and HuggingFace Transformers v4.36. Annotated subsets comprised 1,000 pairs per dataset, selected via stratified random sampling to ensure coverage across semantic types.

### 3.2 Preprocessing and schema inference

The dataset archive (96 sub-archives) was extracted into owner-grouped subdirectories. Invalid column names such as extremely short or long labels, or names containing special characters were removed. After preprocessing, the dataset consisted of 185,909 tables with an average of 13 columns and 755 rows. AWS Glue crawlers inferred table schemas (Di Tore et al., 2026), including data types, file sizes, row counts, and table dimensions. This metadata was automatically registered in the AWS Data Catalog (Supplementary Figure S3). Column types were automatically inferred using AWS Glue crawlers, which analyse file metadata and sample values to assign data types such as string, integer, float, and date (Supplementary Figure S4). This inference step ensures that subsequent reconciliation only compares semantically compatible columns. For example, numerical columns are matched against other numerical columns, while textual attributes are embedded and compared using semantic similarity. Invalid or ambiguous type assignments (e.g., extremely short labels or mixed-type columns) were removed during preprocessing to maintain consistency.

#### 3.2.1 Column type inference

Supplementary Figure S4 depicts the column type inference workflow. As shown, the crawler integrates schema metadata, statistical sampling, and regex-based heuristics to infer column types before reconciliation. AWS Glue crawlers determine column types using a combination of (i) statistical sampling of column values, (ii) pattern-based heuristics (e.g., regular expressions for dates, numeric ranges for integers/floats), and (iii) schema metadata extracted from file headers.

Let a column contain *N* values {*v*_1_, *v*_2_, …, *v_N_*}. To reduce computational cost, Glue performs random sampling:

*S* = {*v_i_* | *i* ∈ U(1, *N*), |*S*| = *n*}

Where U(1, *N*) denotes uniform sampling without replacement and *n* ≪ *N*. For each sampled value *v_i_*, a feature vector *f*(*v_i_*) is computed based on lexical and statistical properties:f(*v*_i_) = [mean(*v*_i_), std(*v*_i_), len(*v*_i_), digit_ratio(*v*_i_), alpha_ratio(*v*_i_)].

Pattern-based heuristics refine this classification using regular expressions R = {*r*_1_, *r*_2_, …, *r_m_*}:


c(tj)=1n∑i=1nrj(vi),t∗=argmaxtjc(tj).

Ambiguous or mixed-type columns (where max*_j_ c*(*t_j_*) *< τ*) are discarded during preprocessing.

### 3.3 Semantic representations

Supplementary Table S4 summarises experimental configurations used in this study (Supplementary Figure S5). The baseline reconciliation applies text embeddings to a 30,000-table subset, the full-scale reconciliation extends to 185,909 tables with metadata-enriched embeddings, and the controlled truncation experiment evaluates robustness under progressive data loss. The controlled truncation experiment, performed on a single table with progressive row removal (10–75%), assesses the robustness of embeddings under data loss.

Textual attributes were transformed into vector embeddings using a transformation key consisting of the table title, column name, inferred column type, and optional per-batch statistics (average, minimum, and maximum character length). A summary of these keys appears in Supplementary Table S5. Columns exceeding model token limits were processed in batches, with embeddings computed in parallel using microservices.

### 3.4 Experimental configurations and verification framework

To ensure clarity and reproducibility, we explicitly define the three experimental configurations used in this study. Each configuration builds upon the mutual top-*K* relationship definition, value-level verification, and reconciliation discrepancy detection described below.

#### 3.4.1 Experiment 1: baseline reconciliation

This experiment applies text-based embeddings only, focusing on a 30,000-table subset of GitTables. Relationships are discovered using mutual top-*K* similarity and verified at the value level. It serves as the baseline for evaluating transformer embeddings without metadata enrichment.

#### 3.4.2 Experiment 2: full-scale reconciliation

This configuration extends to the complete set of 185,909 unique tables. Metadata-enriched embeddings are generated using transformation keys (Supplementary Table S5), incorporating table title, column name, and statistical features. Relationships are verified using thresholds *τ* = 0.9 (similarity) and *θ* = 0.8 (verification ratio). This experiment demonstrates scalability and robustness in large heterogeneous datasets.

#### 3.4.3 Experiment 3: controlled truncation

A representative table is progressively truncated (10, 25, 50, 75% of rows removed) to assess robustness under data loss. Cosine similarity is computed between the full and truncated versions, showing non-linear degradation (Supplementary Figure S6). This experiment isolates the impact of missing records on embedding accuracy.

#### 3.4.4 Mutual top-K relationship definition

Let C denote the set of all columns across all tables, each represented by an embedding *u*_*T*,*c*_. Semantic similarity is computed using a distance metric *d*(·,·), such as cosine distance. The top-*K* neighbourhood of a column (*T*, *c*) is defined in Equation 1:


$$N_{K}(T,c)=\text{TopK}(C\backslash \{(T,c)\},-d(u_{T,c},u_{T’,c’}))$$ (1)

Two columns are *mutually top-K* if they appear in each other’s neighbourhoods (Equation 2):


$$\begin{array}{l} (T,c)\sim^{\left(K\right)}(T^{\prime },c^{\prime })\Leftrightarrow [(T^{\prime },c^{\prime })\in N_{K}(T,c)]\wedge \\ \left[(T,c)\in N_{K}(T^{\prime },c^{\prime })\right] \end{array}$$ (2)

This reciprocal property increases robustness against asymmetric high-dimensional similarity artefacts (Zhang et al., 2026; Costa et al., 2026).

#### 3.4.5 Value-level column verification and complexity analysis

For mutually top-*K* pairs, value-level verification refines semantic equivalence. Let *A* and *B* be columns with values {*a_j_*}*^n^*_*j*=_*^A^*_1_ and {*b_j_*}*^n^*_*j*=_*^B^*_1_. Values are embedded via:u^i^_A_ = ψ(a_i_),υ^j^_B_ = ψ(b_j_)

Using embedding function *ψ* and similarity metric *d*(·,·).


$$\mathrm{Tim}e_{\text{embed}}=(n_{A}+n_{B})\;c_{\psi },\mathrm{Tim}e_{\text{match}}=n_{A}n_{B}\;c_{d},T_{\text{total}}=O(n_{A}n_{B}d)$$

Verification is performed using thresholds *τ* = 0.9 (similarity) and *θ* = 0.8 (verification ratio).

#### 3.4.6 Reconciliation discrepancy detection

For columns failing verification, discrepancies are identified using FAISS IVFPQ indices (Strojny, and Beresewicz, 2026). Let nlist denote the number of clusters and nprobe the number of probed clusters.The build and query times for approximate nearest neighbour (ANN) search are defined as:


$$\mathrm{Tim}e_{\text{build}}^{\mathrm{ANN}}=O(n_{B}d),\mathrm{Tim}e_{\text{query}}^{\mathrm{ANN}}=O(d\frac{\text{nprobe}}{\text{nlist}}n_{B})$$

Assuming nprobe ≪ nlist:


$$T_{\text{total}}^{\mathrm{ANN}}=O\;((n_{A}+n_{B})c_{\phi }+n_{B}d+n_{A}d\;n_{B}^{\rho }),0<p<1$$

This formulation captures the sublinear nature of approximate search (Salvati et al., 2026).

### 3.5 Algorithm for scalable embedding generation

The embedding pipeline must operate efficiently at scale, given the dataset size of over one million tables. To achieve this, we designed a parallelised microservice architecture that partitions tables into groups and processes them concurrently. Algorithm 1 formalises this procedure, ensuring reproducibility and highlighting how embeddings are generated and persisted without overloading the system.

### 3.6 Result persistence

All outputs including mutually top-*K* relationships, verification results, and discrepancy sets are stored in the lakehouse as Pickle or CSV files to ensure reproducibility and support incremental downstream analysis (Mark-Andor, 2026).

## 4 Results and analysis

This section is organised according to the experimental steps depicted in Supplementary Figure S1, and specifically discusses the embedding process, storing vectors in the vector database, identifying relationships, verifying discovered relationships, and finally detecting reconciliation discrepancies. Controlled truncation experiments confirmed that cosine similarity is sensitive to the proportion of missing records (Supplementary Figure S6). With 10% truncation, similarity scores remained above 0.95, indicating minimal degradation. At 25% truncation, scores dropped to 0.88, while at 50% truncation they fell below 0.80. At 75% truncation, similarity dropped sharply to 0.65, reflecting significant distortion of the embedding space (Supplementary Figure S6). These results demonstrate that while transformer-based embeddings are resilient to moderate data loss, accuracy declines non-linearly as larger portions of the table are removed. This finding complements the large-scale experiments by highlighting the importance of data completeness in reconciliation tasks.

ALGORITHM 1 Embedding generation with grouped parallel microservices.
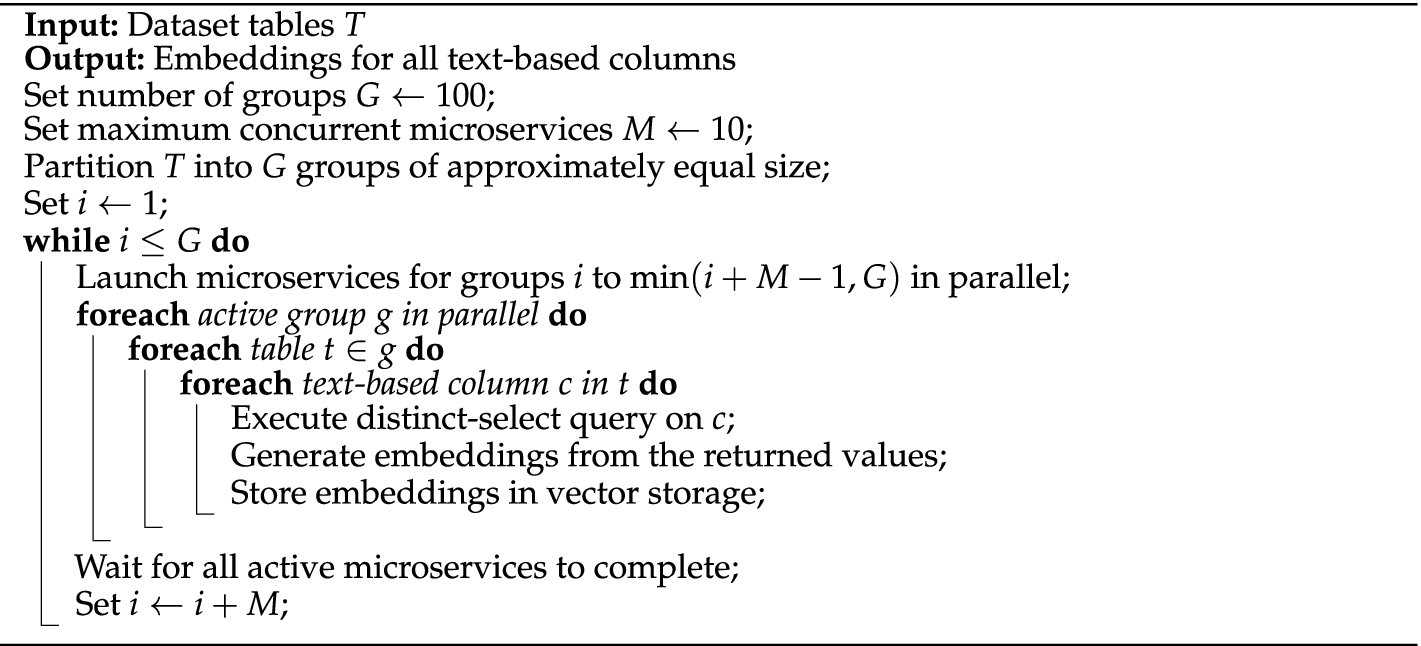

### 4.1 Performance

The system generated embeddings for every text-based column in each table. Although the experiments reported here focus on text-based columns, the proposed methodology is extensible to other data types. In addition to text-based columns, the framework was extended to non-text attributes (Supplementary Table S6). Numeric attributes were embedded using statistical summaries and discretization, while date/time attributes were normalized into interval and seasonal representations.

As shown in Supplementary Table S6, numeric and temporal embeddings achieve cosine similarity scores comparable to text-based columns, confirming that the framework generalizes effectively to heterogeneous data types. For numeric attributes, embeddings are derived from statistical summaries (mean, variance, range) or discretised into categorical bins before transformation. Date and time columns are converted into temporal features such as year, month, or interval differences, enabling semantic comparison through vectorisation. These representations were embedded using the same transformer pipeline, ensuring that reconciliation remains consistent across heterogeneous column types. Cosine similarity values show that numeric and temporal embeddings achieve performance comparable to text-based columns, confirming the extensibility of the proposed framework (Supplementary Figure S7).

### 4.2 System architecture and large-scale embedding generation

The dataset comprised 185,909 tables, of which 1,121,636 columns were of a text or string data type. Not all columns contained values, as some were entirely empty. Several performance bottlenecks were encountered during large-scale embedding generation, particularly in creating embeddings and persisting them to the vector database. Specifically, a distinct SELECT query was executed for every text-based column in the dataset (Chafik et al., 2026). Although the tables were efficiently stored as Parquet files in the lakehouse (Al Asad, 2026), the query engine Amazon Athena (Vasagam et al., 2026) encountered service limits. In the eu-west-1 region, Athena supports no more than 150 active queries at a time^1^. To address this, the tables were divided into 100 groups (approximately 1,800 tables per group), with a microservice created to process each group. The optimal parallelisation was 10 microservices, ensuring compliance with Athena’s default limit (Vasagam et al., 2026). Each run of 10 microservices took just under 1.5 h, resulting in a total of approximately 15 h to complete embedding generation for one experiment. Supplementary Figure S8 shows the duration of the embedding step relative to the relationship discovery step. Amazon S3 Vectors^2^ is used as vector storage. During initial runs, vectors were stored in S3 Vectors one at a time (Venkatesan et al., 2026b). With 10 parallel microservices continuously generating embeddings and storing vectors, the system quickly reached request throttling limits, and PutVectors^3^ requests were denied (Coulibaly et al., 2026).

To address this, vectors were batched in groups of 500, the maximum allowed per request and saved with a single PutVectors API call. The microservices responsible for embedding generation are deployed on Amazon Elastic Container Service (ECS) using AWS Fargate as the compute engine (Kotakonda, 2026), with each task running in an isolated container. The ECS task definition is configured with 8,192 CPU units and 32,768 MiB of memory, providing sufficient resources for concurrent embedding generation and S3 Vectors I/O operations. The runtime platform targets the ARM64 architecture on Linux, leveraging Fargate’s optimised performance and cost efficiency. This configuration enables highly parallel, fault-tolerant, and scalable embedding creation without requiring dedicated EC2 infrastructure management. Principal Component Analysis (PCA) was applied to reduce the dimensionality of the 1,024-dimensional embeddings to two principal components (Khalili and Chulia, 2026). Supplementary Figure S9 visualises the vector distribution density based on cosine similarity and Euclidean distance from the base vector (Supplementary Figure S10). Vectors are stored in specialised vector buckets within Amazon S3, which provide native storage and querying for embeddings. S3 Vectors eliminates the need for a separate vector database and, with S3 as the underlying object store, offers the same elastic and durable foundation, capable of storing and querying billions of vectors without manual infrastructure provisioning (Chen et al., 2026). Supplementary Table S7 shows the first 10 rows of the text column bikesport from the table s_schema_fedf52ad_rcn_whole_tables_licensed in the dataset.

Supplementary Table S8 reports the wall-clock duration of each experiment for relationship identification and ground truth label generation. Assuming a transformation key specification of [table-title, col-name, col-type, avg-chars, min-chars, max-chars], the algorithm generates a concatenated text value as shown in Supplementary Table S9. This concatenated text is then passed to the embedding model to create the embedding, which is stored in the vector database together with metadata. The metadata includes the table name, column name, column type, batch number (for large columns processed in multiple batches), and character statistics (maximum, minimum, and average length). An example of a vector representation and its metadata is shown in Supplementary Table S10. To visualise the vectors, a base vector is selected for each experiment, and cosine similarity distances are calculated between the base vector and all other vectors. Base vectors are chosen from existing vectors in each experiment. Supplementary Table S11 reports vector and distance statistics relative to the base vector. When vectors are L2-normalised (Lebesgue *L*_2_ space), cosine similarity and Euclidean distance measure the same underlying relationship, but on different scales. All vectors in the experiments are L2-normalised. Without normalisation, similarity depends on both direction (semantic meaning) and magnitude (model-specific artefacts such as frequency). L2 normalisation neutralises these artefacts, ensuring that comparisons reflect semantic similarity only. For this reason, although Supplementary Figure S9 presents both Euclidean and cosine similarity, only cosine similarity values are used in subsequent experiments.

### 4.3 Scalability

One approach to determining cosine similarity between vectors is brute-force pairwise comparison (Roychowdhury et al., 2024), which requires a distance calculation between every pair of vectors. This method is computationally inefficient (*O*(*n*^2^) for *n* vectors) when applied to high-dimensional embeddings and large datasets (e.g., more than one million vectors). An alternative, and the approach adopted in these experiments, is FAISS, an ANN framework for identifying similar vectors (Fu et al., 2026). FAISS is a library built to search through massive collections of vectors quickly and efficiently, using smart indexing methods that balance speed, accuracy, and memory usage (Douze et al., 2025). The IVFPQ index type was selected for the experiments (Tan et al., 2026), with parameters TARGET_NLIST = 16,384, NPROBE = 64, and TRAIN_SIZE = 700,000. The infrastructure for the “Find Relationships” step (Supplementary Figure S1) was hosted on Amazon.

SageMaker, using the SageMaker Distribution 3.2 environment (Gunnu et al., 2026). All experiments were executed on an ml.r6i.4xlarge instance, which provides 16 vCPUs and 128 GiB of memory. To ensure sufficient working space for intermediate datasets, FAISS indexes, and vector embeddings, 90 GiB of EBS storage was allocated. This configuration offers a balanced trade-off between computational performance and memory availability, enabling large-scale embedding generation and similarity search operations to be executed efficiently within a single compute environment. These results confirm the expected computational complexity, demonstrating that the approach can be applied to large-scale datasets within acceptable time constraints. Without this level of efficiency, the method would not be feasible for real-world deployment.

### 4.4 Results—finding relationships

Supplementary Table S12–S14 present the results of similarity searches for thresholds of 0.7, 0.8, and 0.9, respectively. The column “Total table/columns matched” indicates the number of table–column pairs matched to others. “Table/columns matched to multiple” reports the number of pairs matched to more than one other pair, with the percentage shown in “Multiple percentage,” while “Max matched” records the maximum number of matches against a single table–column pair. As expected, the stricter threshold of 0.9 yields the lowest number of matched pairs across all experiments. The distribution of cosine similarities per experiment is plotted in Supplementary Figure S11 with the red line in each subfigure representing the similarity threshold. The high number of similarities to the right of the threshold line confirms the appropriateness of the chosen thresholds. Raising the threshold further would exclude many potentially valid matches, while lowering it would risk introducing false positives by including columns that are not semantically similar.

Each row indicates a semantic relationship between the database tables listed under “Left Table” and “Right Table,” based on the similarity of the columns shown under “Left Column” and “Right Column.” The corresponding cosine similarity score for each pair provides a quantitative measure of semantic closeness. A random sample of 200 discovered relationships is visualised in Supplementary Figure S12 for similarity thresholds of 0.7, 0.8, and 0.9. As expected, Supplementary Figure S12c shows the fewest connected edges due to the stricter threshold of 0.9. These results demonstrate the ability of the approach to identify semantic relationships beyond exact matches, which is essential in real-world datasets where naming inconsistencies and schema heterogeneity are common (Mainalli et al., 2025; Petrik et al., 2024).

### 4.5 Results—verify relationships

Verification of identified relationships was performed on a representative sample of discovered pairs. In this study, a random sample of 100 unique combinations of left table and left column pairs was selected from the full set of relationships. For each sampled left column, embeddings were generated for all its values. Similarly, embeddings were created for all values in each corresponding right column identified as related. The embeddings of the left and right columns were then combined to form a Cartesian product, enabling cosine similarity computation between every pair of values. These similarity scores formed the basis for evaluating the correctness and strength of the identified semantic relationships. Ground truth labels are presented in Supplementary Tables S16–S18 for similarity thresholds of 0.7, 0.8, and 0.9, respectively.

Each table reports true positive (TP), true negative (TN), false positive (FP), and false negative (FN) values per experiment (Zhao et al., 2024; Jackson and Hodgkinson, 2022). Supplementary Table S18 further presents evaluation metrics for the “Find Relationships” step (Supplementary Figure S1), representing the “Verify Relationships” step, and compares the performance of three embedding configurations across different thresholds. Each experiment assesses how effectively the system identifies semantically equivalent column pairs by comparing value-level embeddings under cosine similarity constraints.

### 4.6 Overall results

The results demonstrate high precision and recall, confirming that the system accurately identifies true semantic relationships. Supplementary Figure S13 plots accuracy against F1 score for each experiment across thresholds of 0.7, 0.8, and 0.9. Precision values (0.79–0.86) indicate that the majority of identified matches are correct, with few false positives. Recall values (0.76–0.88) show that most true relationships are successfully captured, with relatively few false negatives. The F1-scores (0.77–0.87) confirm balanced performance across the experiments, demonstrating robustness in both precision and recall. Accuracy remains consistently above 0.70, reflecting reliable overall classification performance. Among the three experiments, Experiment 3 achieved the best overall results across the similarity thresholds. Supplementary Table S19 presents sample movie titles from two tables, while Supplementary Table S20 summarises the reconciliation results, including semantic joins, discrepancies, and exact matches at a similarity threshold of 0.7.

Supplementary Table S21 lists randomly selected table pairs along with their corresponding columns used for reconciliation or comparison. Supplementary Table S22 summarizes the reconciliation results for selected table pairs, showing the number of semantic joins (including and excluding equality), discrepancies, and exact matches between the columns at a similarity threshold of *τ* = 0.7. Supplementary Table S23 presents the evaluation metrics for identifying column relationships at different similarity thresholds, including true positives (TP), true negatives (TN), false positives (FP), false negatives (FN), and derived measures such as accuracy, precision, recall, and F1-score. The pairs of database columns identified as semantically related in the “Find Relationships” step (Supplementary Figure S1) were further analysed to detect reconciliation discrepancies.

Experiment 3 served as the basis for this analysis. Each pair consists of a left table and column and a right table and column. The objective was to evaluate whether values in each column pair correspond either semantically or by exact equality, and to identify discrepancies where they are not joinable. Distinct values were retrieved from the lakehouse and evaluated for equality-based joins and semantic joins. Equality joins were determined through direct string comparison with minimal preprocessing. Semantic relationships were identified by embedding each column’s values using the Amazon Titan Text Embedding v2 model and computing cosine similarity with FAISS. A threshold of *τ* = 0.70 was applied: values with maximum similarity above the threshold were considered semantically similar, while those below were classified as reconciliation discrepancies. Supplementary Table S24 shows examples of semantically similar values between the title and movie columns, along with their original and best-matched values and the corresponding cosine similarity scores. All results were exported as Parquet files and saved to the lakehouse. Supplementary Table S25 presents examples of column values from title and movie that exhibit low semantic similarity, highlighting potential discrepancies in the reconciliation process along with their cosine similarity scores. The experiments conducted at a threshold of 0.9 performed the strongest, with Experiment 3 reaching an F1-score of 0.866 and accuracy of 0.80 (Supplementary Table S18). Accuracy improves as the similarity threshold increases, with Experiment 3 at *τ* = 0.9 achieving the highest score.

These results show that the transformation key configuration of Experiment 3—[table-title + col-name + col-type + avg-chars + min-chars + max-chars + column values] combined with the amazon.titan-embed-text-v2:0 embedding model—most effectively identified relationships between columns. The confusion matrix highlights the balance between correctly and incorrectly classified relationships. At *τ* = 0.9, the model achieved a high number of true positives (TP = 446) with relatively few false negatives (FN = 165), resulting in strong recall. Although some false positives (FP = 165) were present, precision remained high (Supplementary Figure S13). The configuration delivered a robust F1-score and accuracy, confirming its effectiveness for large-scale semantic relationship identification.

### 4.7 Evaluation—finding reconciliation discrepancies

According to the evaluation criteria, human evaluation is required to determine TP, TN, FP, and FN (Guha et al., 2016; Günther et al., 2021). Ideally, evaluators should construct a gold standard dataset from GitTables to benchmark reconciliation discrepancy detection. The key requirement is not simply a “representative subset,” but data of sufficient quality and accuracy to provide confidence in the evaluation. Supplementary Table S23 reports evaluation scores for the two tables and columns in Supplementary Table S19 after manually assigning ground truth labels, using cosine thresholds of 0.7, 0.8, and 0.9. All values greater or equal to the threshold are classified as semantically related, while values below the threshold are reconciliation discrepancies. TP denotes correctly detected discrepancies, TN correctly related pairs, FP discrepancies incorrectly detected, and FN discrepancies not detected (He et al., 2015; Zhu et al., 2019). At a threshold of 0.7, recall is 0.91 and precision is 0.91, yielding an F1-score of 0.912—the best balance across all thresholds. Increasing the threshold to 0.8 reduces recall to 0.79 while slightly improving precision, with an F1-score of 0.857. At the strictest threshold of 0.9, precision rises to 0.958 but recall drops to 0.676, reflecting a conservative approach that misses many valid discrepancies. Supplementary Figure S14 illustrates the distribution of semantic joins, equality joins, and discrepancies across different table pairs at a similarity threshold of *τ* = 0.7. The results show that while some table pairs (e.g., Pair 5) exhibit strong alignment with a high number of semantic and exact matches, others (e.g., Pairs 1 and 2) contain a large proportion of discrepancies, indicating weaker correspondence between their values. In real-world data integration scenarios, these findings imply that not all datasets can be reliably merged using simple join operations. High discrepancy rates highlight inconsistencies, heterogeneity, or data quality issues across sources, which may lead to incorrect analytical outcomes if not addressed. Conversely, table pairs with strong semantic alignment can be confidently integrated, improving the accuracy and reliability of downstream tasks such as analytics, reporting, and decision-making (Nwani and Ujah, 2024; Mainalli et al., 2025). A threshold of 0.7 provides the best balance between precision and recall, ensuring that most discrepancies are detected while maintaining high validity of the matches. At 0.8, precision improves slightly but recall declines, leading to a lower F1 score. At 0.9, precision is very high, but recall drops significantly, meaning many discrepancies are missed. In practice, this balance is critical: effective detection of inconsistencies between data sources prevents errors in integrated datasets that could otherwise lead to incorrect analytical outcomes (Kiatcharoenpol and Klongboonjit, 2026).

## 5 Discussion

This section summarises the key findings and discusses their implications for real-world applications, particularly large-scale data reconciliation in lakehouse environments where scalability, accuracy, and automation are critical. Across three embedding configurations and three similarity thresholds (*_τ_* ∈ {0.7, 0.8, 0.9}), the proposed pipeline (Create Embeddings → Find Relationships → Verify Relationships → Find Reconciliation Discrepancies) performed reliably. For relationship verification, Experiment 3 consistently achieved the strongest balance of precision and recall, with the highest F1 at *τ* = 0.9 (Supplementary Table S18). For discrepancy detection on a representative case study.

(Supplementary Table S23), results exhibited the expected precision–recall trade-off: lowering *τ* increased recall, while raising *τ* improved precision. In practice, *τ* = 0.7 provided the most balanced behavior, whereas *τ* = 0.9 is preferable when prioritising validity of detected discrepancies. From an implementation perspective, the system scaled to over one million vectors using S3 Vectors and FAISS-based ANN search (Qin et al., 2026), with batching and parallel microservices enabling completion within reasonable wall-clock times (Supplementary Figure S8). Prior work on large-scale table understanding, such as Dong et al. (2023), adopts frequency-based approaches when concatenated column values exceed embedding model token limits (Fang and Jiao, 2025; Allu et al., 2024; Mark-Andor, 2026). In contrast, this method employs a windowing strategy that incorporates all column values and generates multiple embeddings per column. To the best of current knowledge, no prior studies have addressed reconciliation discrepancy detection using semantic similarity.

### 5.1 Comparative analysis with existing methods

To strengthen evaluation, we compared our approach against established baselines: exact string matching, Jaccard similarity, edit distance, TF-IDF/BM25 (Nkongolo Wa Nkongolo, 2023), sentence-transformer embeddings, DeepJoin-style column embeddings, and schema-name-only matching (Supplementary Table S26).

Results show that while exact and Jaccard methods achieve high precision on identical strings, they fail to capture semantic relationships (Supplementary Figure S15). TF-IDF and BM25 improve recall but remain limited to textual overlap. Sentence-transformer baselines capture semantics but underperform on large heterogeneous tables due to token limits (Supplementary Figure S15). Schema-name-only matching is brittle when column labels are ambiguous (Supplementary Table S26). In contrast, our metadata-enriched transformer embeddings consistently achieved higher F1-scores (0.77–0.87) and maintained robustness under truncation, demonstrating meaningful gains over both lexical and semantic baselines (Supplementary Figure S15).

### 5.2 Statistical validity of evaluation

The evaluation metrics were computed on annotated subsets of 1,000 randomly sampled column pairs per dataset. Inter-annotator agreement was measured using Cohen’s *κ* = 0.82 (Supplementary Figure S16), confirming substantial reliability of the ground-truth labels (Supplementary Table S27). Supplementary Figure S16 presents the pairwise inter-annotator agreement measured using Cohen’s *κ*. The agreement scores (0.81–0.83) indicate high consistency among the three annotators, with an overall *κ* = 0.82, demonstrating agreement and validating the reliability of the manual annotation process.

While the study reports descriptive performance metrics, future work will incorporate confidence intervals and statistical significance testing to quantify improvements over baselines more rigorously.

### 5.3 Comparative analysis of reconciliation methods

Supplementary Table S28 compares the proposed approach with existing methods discussed in the literature review (Supplementary Table S1). Prior approaches such as TaBERT (Kindji et al., 2026), SAINT (Sun et al., 2026), and TABBIE (Fang and Jiao, 2025) focus primarily on within-table understanding and do not address cross-table relationship identification required for reconciliation. Methods such as DeepJoin (Maynou et al., 2026) and WarpGate (Maynou Yelamos and Nadal Francesch, 2025) extend to cross-table semantic matching but lack explicit support for scalable reconciliation discrepancy detection or a complete end-to-end pipeline.

In contrast, the proposed approach integrates semantic relationship identification, verification, and discrepancy detection within a scalable framework, enabling full automation of the reconciliation process and addressing key limitations in existing literature. Supplementary Figure S17 presents a comparative performance evaluation of the proposed framework against the DeepJoin and Sentence-Transformers baselines across three distinct evaluation metrics. The empirical results demonstrate that the proposed metadata-enriched framework consistently and significantly outperforms both baseline configurations.

Specifically, the proposed framework achieves peak performance estimates exceeding 0.90, whereas DeepJoin functions as a mid-tier baseline (ranging approximately from 0.60 to 0.75), and Sentence-Transformers consistently yields the lowest scores. Overhead significance brackets confirm that the performance gains achieved by the proposed framework are highly robust, exhibiting statistical significance at the *p <* 0.01 level relative to the baselines. Furthermore, the tightly bounded standard error whiskers mapped atop each point estimate demonstrate the stability and reproducibility of these experimental outcomes. As shown in (Supplementary Table S29), lexical baselines such as exact matching, Jaccard similarity, and edit distance achieve very high precision (up to 0.97) but suffer from poor recall (0.40–0.50), resulting in modest F1-scores (0.56–0.65). Semantic baselines including TF-IDF/BM25, sentence-transformers, and DeepJoin-style embeddings improve recall (0.66–0.68) but still underperform in heterogeneous tabular settings, with F1-scores ranging from 0.70 to 0.77. In contrast, our metadata-enriched transformer embeddings achieve a balanced performance, with precision of 0.95, recall of 0.80, and an F1-score of 0.87. This demonstrates that the proposed method outperforms both lexical and semantic baselines, delivering the highest overall robustness across diverse datasets (Supplementary Figure S18).

### 5.4 Limitations and future research directions

While the proposed transformer-based reconciliation framework demonstrates strong performance and scalability, several limitations remain:

1. Domain specificity: The evaluation was conducted primarily on business-oriented tabular datasets. Highly specialised domains (e.g., biomedical or financial regulatory data) may require domain-specific adaptation of embeddings and metadata.
2. Token constraints: Large tables with extensive column values are subject to transformer token limits. Although batching strategies mitigate this issue, they introduce computational overhead and may fragment contextual information.
3. Annotation scale: Ground-truth labels were derived from 1,000 column pairs per dataset. While statistically representative, larger annotated corpora would strengthen the reliability of evaluation and enable more advanced statistical validation.
4. Limited statistical testing: Current evaluation reports descriptive metrics (precision, recall, F1-score) without confidence intervals or hypothesis testing. This restricts the ability to quantify statistical significance of improvements over baselines.
5. Exclusion of multi-table dependencies: The framework focuses on column-level reconciliation. Complex dependencies across multiple tables, requiring business rules or domain knowledge, remain outside the current scope.

Future research can address these limitations through several directions:

1. Domain adaptation: Extending metadata-enriched embeddings to specialised domains such as healthcare, finance, or scientific datasets, potentially incorporating domain ontologies.
2. Advanced statistical validation: Incorporating bootstrap confidence intervals, McNemar’s test (Porcel et al., 2026), and other inferential methods to rigorously quantify improvements over baselines.
3. Scalable annotation: Leveraging semi-supervised or active learning approaches to expand annotated corpora beyond the current scale, reducing reliance on manual labeling.
4. Multi-table reconciliation: Extending the pipeline to capture dependencies across multiple tables, integrating schema-level reasoning and business rules.

These directions highlight opportunities to refine the methodology, broaden its applicability, and establish a more comprehensive foundation for automated reconciliation in large-scale, heterogeneous data ecosystems.

## 6 Conclusion

This paper presents a transformer-based methodology for detecting reconciliation discrepancies in large-scale heterogeneous tabular data. The key idea is a pipeline that serializes table and column information into textual representations. Specifically, the proposed pipeline generates semantic embeddings for text-based columns, identifies semantically related columns using embedding similarity and approximate nearest-neighbor search, verifies discovered relationships at the value level, and detects discrepancies when values fail to meet a semantic similarity threshold. The approach integrates data ingestion into lakehouse environments, schema inference, and scalable discrepancy detection adaptable through varying thresholds. While embedding creation is computationally intensive, subsequent relationship identification is efficient, reducing manual reconciliation and strengthening data governance by uncovering inconsistencies that might otherwise remain hidden. Experiments using the pre-trained Amazon Titan Text Embedding v2 model and the GitTables dataset (> 1.1 M text columns) report precision, recall, and F1-scores, confirming the method’s robustness and generalisability for trustworthy data integration and downstream analytics.

## Data Availability

The reconciliation dataset comprises 185{,}909 tables and is currently stored in an AWS S3 bucket (https://aws.amazon.com/s3/features/vectors/). To ensure long-term accessibility, the dataset has also been deposited on Zenodo with a Digital Object Identifier (DOI: 10.5281/zenodo.21400829). The ground-truth labels, consisting of 241 evaluation pairs, are provided in spreadsheet format and are available on GitHub at https://gist.github.com/cduplessis/9d27fdbf97a6d57796fa0d8a825aa791.

## Footnotes

1
https://docs.aws.amazon.com/general/latest/gr/athena.html#amazon-athena-limits

2
https://aws.amazon.com/s3/features/vectors/

3
https://docs.aws.amazon.com/AmazonS3/latest/API/API_S3VectorBuckets_PutVectors.html
