# Correction: Detecting reconciliation discrepancies in tabular data using transformers

**DOI:** 10.3389/frai.2026.1977222

**Published:** 2026-10-13

**Authors:** Carl Du Plessis, Mike Wa Nkongolo

**Affiliations:** 1 Department of Computer Science, University of Pretoria, Pretoria, South Africa; 2 Department of Informatics, University of Pretoria, Pretoria, South Africa

**Keywords:** data lakes, discrepancy detection in tabular datasets, entity matching, semantic embeddings, transformer models

Supplemental material “Supplementary File 1.zip” was omitted. The file(s) have now been published.

The original version of this article has been updated.

