# Supplementary material for: Detecting reconciliation discrepancies in tabular data using transformers
Source: Front Artif Intell. 2026 Aug 24;9:1840051. doi: 10.3389/frai.2026.1840051 (PMC13547505; doi:10.3389/frai.2026.1840051)
Supplement: Supplementary file 1 [file Table_1.docx]

| **Type** | **Reference** | **Description** |
| --- | --- | --- |
| Tables as a collection of sentences | Deng et al. (2022) | A Transformer encoder tailored for relational databases that captures row and column awareness. |
| Tables as a collection of sentences | Yin et al. (2020) | TaBERT flattens a table into a sequence so that the Transformer can process it similarly to a sentence, rather than treating rows and columns separately. |
| Tables as a collection of sentences | Yu et al. (2021) | GraPPa is a grammar-augmented pre-training method for table semantic parsing. It constructs a synchronous context-free grammar (SCFG) from text-to-SQL datasets to capture common question-SQL patterns. |
| Tables as a collection of sentences | Somepalli et al. (2021) | SAINT is a row-column attentive Transformer with enhanced embeddings that performs attention across both dimensions of a table. |
| Tables as a collection of sentences | Herzig et al. (2020) | TAPAS (Table Parser) is a supervised model for table-based question answering that performs reasoning directly over tabular data without generating intermediate logical representations. |
| Tables as a collection of sentences | Iida et al. (2021) | TABBIE (Tabular Information Embedding) uses a dual-Transformer design in which one Transformer processes rows and another processes columns independently. Their encoded representations are merged through pooling operations at each layer. |
| Tables as a collection of sentences | Glass et al. (2021) | RCI (Row and Column Intersection) is a table question-answering method that estimates answer likelihoods separately for rows and columns and combines them at their intersection to identify the most probable answer cell. |
| Tables as table (does not treat a table as a sentence) | Arik and Pfister (2021) | TabNet is a deep learning architecture designed specifically for tabular datasets. It uses a sequential attention mechanism to select semantically important feature subsets at each decision step. |
| Extract features as sentences | Koloski et al. (2025) | TabPTM is a pre-trained model for tabular data that creates row embeddings for classification and regression tasks. It embeds rows into a shared space and computes distances to k-nearest neighbours. |
| Table rows as a collection of sentences | Ye et al. (2025) | Dataset features are serialized into textual prompts of the form “This {column} is {value}” to generate LLM-based embeddings. Although this approach improves accuracy compared with competing models, it introduces computational overhead for large datasets because an embedding must be generated for each value in every column (Koloski et al., 2025). |
| Columns and rows as a collection of sentences | Our research | The proposed approach generates embeddings for all column values enriched with metadata such as table title, column name, data type, and statistical characteristics. To handle large tables, values are split across multiple embeddings when token limits are reached. For cross-table comparisons, embeddings are generated not only for the target column value but also for related values within the same row, providing richer contextual information. |

**TABLE S1– Transformer based models for encoding tabular data.**

| **Statistic** | **Value** |
| --- | --- |
| Parquet files | 1,000,000 |
| Average columns per table | 12 |
| Average rows per table | 142 |
| Annotated tables (column) | 723K (DBpedia), 738K (Schema.org) |
| Number of unique semantic types | 835 (DBpedia), 677 (Schema.org) |

**Table S2. GitTables dataset statistics (**<https://doi.org/10.5281/zenodo.21400829>**).**

| **Table Domain** | **10%**  **Removed** | **25%**  **Removed** | **50%**  **Removed** | **75%**  **Removed** |
| --- | --- | --- | --- | --- |
| Financial Records | 0.96 | 0.89 | 0.81 | 0.64 |
| Healthcare Dataset | 0.95 | 0.87 | 0.79 | 0.66 |
| E-commerce Transactions | 0.94 | 0.88 | 0.80 | 0.65 |
| Social Media Profiles | 0.95 | 0.86 | 0.78 | 0.63 |
| Scientific Measurements | 0.96 | 0.90 | 0.82 | 0.67 |

**TABLE S3. Cosine similarity under progressive truncation across multiple representative tables.**

| **Experiment** | **Dataset** | **Evaluation** |
| --- | --- | --- |
| Baseline Reconciliation | 30,000 tables | Relationship discovery |
| Full-Scale Reconciliation | 185,909 tables | Scalability and robustness |
| Controlled Truncation | 1 table (10% to 75% of rows removed) | Robustness under data loss |

**TABLE S4. Summary of experimental configurations.**

| **Transformation Key** | **Description** |
| --- | --- |
| table-title | Source table name |
| col-name | Column name |
| col-type | Column data type |
| avg-chars | Average character length per batch |
| min-chars | Minimum character length per batch |
| max-chars | Maximum character length per batch |

**TABLE S5. Transformation keys for embedding metadata.**

| **Data Type** | **Embedding Approach** | **Cosine Similarity** |
| --- | --- | --- |
| Text Columns | Transformer-based contextual embeddings | 0.93 |
| Numeric Attributes | Statistical summary + binning embeddings | 0.90 |
| Date/Time Attributes | Normalized interval/seasonal embeddings | 0.88 |
| Mixed Columns (Text + Numeric) | Hybrid concatenated embeddings | 0.91 |

**TABLE S6. Embedding performance for non-text data types compared to text-based columns.**

| **Row** | **License** | **First Name** | **Last**  **Name** | **City** | **State** | **Time** | **Team/Sponsor** |
| --- | --- | --- | --- | --- | --- | --- | --- |
| 1 | 277676 | Michael | Vasey | Flower Mound | TX | 1:26:51.00 | BikeSport |
| 2 | 188575 | Nathan | Winkelmann | Huntsville | TX | 1:27:45.00 | Bike |
| 3 | 111415 | Jeffrey | Shoemake | Houston | TX | 1:30:28.00 | EnviroInks.com/Bike |
| 4 | 165778 | Kyle | Johnson | Denver | CO | 1:31:38.00 | Matrix/RBM |
| 5 | 0 | Chris | Moore | – | – | 1:32:10.00 | Thule |
| 6 | 270946 | Jason | Krempin | Austin | TX | 1:33:13.00 | Ozone |
| 7 | 264098 | Ian | Fleming | Damon | TX | 1:34:12.00 | Cycle |
| 8 | 0 | Tom | Stolz | – | – | 1:34:36.00 | OMW |

**TABLE S7. Values from table : s_schema_fedf52ad_rcn_whole_tables_licensed, column : bikesport.**

| **Experiment** | **Find Relationships**  **Duration**  **(hh:mm:ss)** | **Evaluation Duration**  **(hh:mm:ss)** |
| --- | --- | --- |
| Exp 1 | 00:10:44 | 02:57:22 |
| Exp 2 | 00:11:10 | 03:05:47 |
| Exp 3 | 00:11:23 | 02:51:08 |

**TABLE S8. Wall clock duration for each experiment- Cosine similarity.**

| **Field** | **Value** |
| --- | --- |
| Table Identifier | fedf52ad_rcn_whole |
| Column | bikesport |
| Data Type | string |
| Average Characters | 45 |
| Minimum Characters | 10 |
| Maximum Characters | 67 |
| Values | 03/01/2010 \| Place, License \| 1,277676, Michael, Vasey, Flower \| 2,188575, Nathan, Winkelmann, Huntsville, TX, 1:27:45.00, Bike \| 3,111415, Jeffrey, Shoemake, Houston, TX, 1:30:28.00, EnviroInks.com/Bike \| 4,165778, Kyle, Johnson, Denver, CO, 1:31:38.00, Matrix/RBM \| 5,0, Chris, Moore, , , 1:32:10.00, Thule \| 6,270946, Jason, Krempin, Austin, TX, 1:33:13.00, Ozone \| 7,264098, Ian, Fleming, Damon, TX, 1:34:12.00, Cycle |

**TABLE S9. Values from table : s_schema_fedf52ad_rcn_whole_tables_licensed, column : bikesport.**

| **Field** | **Value** |
| --- | --- |
| vector | [-0.0509, 0.0062, 0.0239, -0.0174, 0.0509, -0.0136, -0.0153, -0.0289, -0.0583, 0.0096, -0.0155, 0.0061, -0.0241, 0.0244, -0.0162, 0.0334, 0.0395, -0.0043, 0.0074, 0.0341, 0.0415, 0.0042, 0.000091, 0.0056, -0.0124, -0.0157, -0.0046, 0.0179, 0.0169, -0.0160, ...] |
| table_title | s_schema_fedf52ad_rcn_whole_tables_licensed |
| column_name | bikesport |
| batch | 2 of 2 |
| max_chars | 69 |
| min_chars | 10 |
| avg_chars | 42 |

**TABLE S10. Example of an embedding including metadata (vector truncated).**

| **Exp** | **Number of Vectors** | **Distance Metric** | **Range**  **(Difference to Base Vector)** | **Mean**  **(Difference to Base Vector)** |
| --- | --- | --- | --- | --- |
| 1 | 1,097,562 | Cosine Similarity | -0.0899 to 1.0000 | 0.1323 |
| 2 | 1,167,000 | Cosine Similarity | -0.0703 to 1.0000 | 0.2360 |
| 3 | 1,141,839 | Cosine Similarity | -0.1102 to 1.0000 | 0.2116 |

**TABLE S11. Vector distance statistics.**

| **Exp** | **Columns Matched** | **Columns Matched to Multiple Columns** | **Multiple Match Percentage** | **Maximum Matches** |
| --- | --- | --- | --- | --- |
| 1 | 453,915 | 231,278 | 46.25% | 12 |
| 2 | 269,842 | 161,007 | 54.83% | 20 |
| 3 | 451,387 | 233,238 | 51.67% | 67 |

**TABLES12. Similarity search results-threshold:0.7.**

| **Exp** | **Columns Matched** | **Columns Matched to Multiple Columns** | **Multiple Match Percentage** | **Maximum Matches** |
| --- | --- | --- | --- | --- |
| 1 | 428,133 | 211,919 | 49.50% | 12 |
| 2 | 250,764 | 147,035 | 58.63% | 20 |
| 3 | 428,997 | 216,459 | 50.46% | 33 |

**TABLES13. Similarity search results-threshold:0.8.**

| **Exp** | **Total Tables/Columns Matched** | **Tables/Columns Matched to Multiple Matches** | **Multiple Match Percentage** | **Maximum Matches** |
| --- | --- | --- | --- | --- |
| 1 | 364,901 | 168,792 | 46.25% | 12 |
| 2 | 189,260 | 103,772 | 54.83% | 20 |
| 3 | 371,561 | 175,644 | 47.27% | 26 |

**TABLES14. Similarity search results-threshold:0.9.**

| **ID** | **Left Table** | **Left Column** | **Right Table** | **Right Column** | **Cosine Similarity** |
| --- | --- | --- | --- | --- | --- |
| 909685 | s_schema_920c45f3_dowjones_parent_tables_licensed | country | s_schema_73d05bb4_bpardee_id_tables_licensed | country | 0.934347 |
| 3839 |  |  |  |  |  |
| 8720 | s_schema_e132c4ab_dfirence_object_tables_licensed | windows.ai.machinelearning.preview.dll | s_schema_9795a799_dfirence_object_tables_licensed | windows.ai.machinelearning.preview.dll | 0.995733 |
| 22000 | s_schema_ea9a519a_ropensci_sampling_rate_tables_licensed | type | s_schema_ea9a519a_ropensci_data_rate_tables_licensed | type | 0.909900 |
| 186946 | s_schema_2ca5cbc2_jernejciczan_half_life_tables_licensed | izdelek | s_schema_9ec8875d_maroskukan_half_life_tables_licensed | product | 0.919053 |

**TABLE S15. Example of joinable columns (Experiment 3).**

| **Experiment** | **N** | **Threshold** | **TP** | **TN** | **FP** | **FN** | **Accuracy** | **Precision** | **Recall** | **F1 Score** |
| --- | --- | --- | --- | --- | --- | --- | --- | --- | --- | --- |
| 1 | 201 | 0.7 | 135 | 0 | 66 | 0 | 0.6716 | 0.6716 | 1.0000 | 0.8036 |
| 2 | 149 | 0.7 | 96 | 0 | 53 | 0 | 0.6443 | 0.6443 | 1.0000 | 0.7837 |
| 3 | 195 | 0.7 | 144 | 0 | 51 | 0 | 0.7385 | 0.7385 | 1.0000 | 0.8496 |
| **Average** | **181.67** | **0.7** | **125.00** | **0.00** | **56.67** | **0.00** | **0.6848** | **0.6848** | **1.0000** | **0.8123** |

**Table S16. Identifying Column Relationships Evaluation (Threshold = 0.7)**

| **Experiment** | **N** | **Threshold** | **TP** | **TN** | **FP** | **FN** | **Accuracy** | **Precision** | **Recall** | **F1 Score** |
| --- | --- | --- | --- | --- | --- | --- | --- | --- | --- | --- |
| 1 | 201 | 0.8 | 131 | 10 | 56 | 4 | 0.7015 | 0.7005 | 0.9704 | 0.8137 |
| 2 | 149 | 0.8 | 94 | 15 | 38 | 2 | 0.7315 | 0.7121 | 0.9792 | 0.8246 |
| 3 | 195 | 0.8 | 138 | 10 | 41 | 6 | 0.7590 | 0.7710 | 0.9583 | 0.8545 |
| **Average** | **181.67** | **0.8** | **121.00** | **11.67** | **45.00** | **4.00** | **0.7307** | **0.7279** | **0.9693** | **0.8309** |

**TABLES17. Identifying column relationships-evaluation (threshold:0.8).**

| **Experiment** | **N** | **Threshold** | **TP** | **TN** | **FP** | **FN** | **Accuracy** | **Precision** | **Recall** | **F1 Score** |
| --- | --- | --- | --- | --- | --- | --- | --- | --- | --- | --- |
| 1 | 201 | 0.8 | 131 | 10 | 56 | 4 | 0.7015 | 0.7005 | 0.9704 | 0.8137 |
| 2 | 149 | 0.8 | 94 | 15 | 38 | 2 | 0.7315 | 0.7121 | 0.9792 | 0.8246 |
| 3 | 195 | 0.8 | 138 | 10 | 41 | 6 | 0.7590 | 0.7710 | 0.9583 | 0.8545 |
| **Average** | **181.67** | **0.8** | **121.00** | **11.67** | **45.00** | **4.00** | **0.7307** | **0.7279** | **0.9693** | **0.8309** |

**TABLE S18. Identifying column relationships- evaluation (threshold: 0.9).**

| **Source Table** | **Column** | **Sample Values** |
| --- | --- | --- |
| s_schema_5253f0f7_olehmberg_thing_tables_licensed | title | *I Am Legend*, *Made of Honor*, *Couples Retreat*, *The Bucket List*, *Mamma Mia!* |
| s_schema_342634fd_amherst_statistics_half_life_tables_licensed | movie | *Django Unchained*, *Parental Guidance*, *The Impossible*, *Jack Reacher*, *This Is 40* |

**TABLE S19. Sample values.**

| **τ (Threshold)** | **Table A Values** | **Table B Values** | **Semantic Joins (Including Equality Joins)** | **Semantic Joins (Excluding Equality Joins)** | **Discrepancies** | **Equality Joins** |
| --- | --- | --- | --- | --- | --- | --- |
| 0.7 | 654 | 609 | 447 | 34 | 207 | 413 |

**TABLE S20. Summary statistics for reconciliation between column pairs.**

| **Table Pair No.** | **Table**  **A** | **Column A** | **Table**  **B** | **Column B** |
| --- | --- | --- | --- | --- |
| 1 | s_schema_162149b7_rcn_whole_tables_licensed | cross | s_schema_29e5d082_rcn_parent_tables_licensed | of |
| 2 | s_schema_164e96ed_hanisaf_then_tables_licensed | requireddate | s_schema_aa7db032_aws_quickstart_then_tables_licensed | requireddate |
| 3 | s_schema_72a9bf57_pranayjagtap_living_thing_tables_licensed | __index_level_3__ | s_schema_730c45ec_pranayjagtap_living_thing_tables_licensed | __index_level_3__ |
| 4 | s_schema_2c8db01b_mslonik_quick_time_tables_licensed | extensions | s_schema_2c8db01b_mslonik_object_tables_licensed | extensions |
| 5 | s_schema_83ac6d15_tedunderwood_then_tables_licensed | htid | s_schema_f12180c3_tedunderwood_parent_tables_licensed | docid |

**TABLE S21. Randomly selected tables.**

| **Table Pair No.** | **Table A Values** | **Table B Values** | **Semantic Joins (Including Equality Joins)** | **Semantic Joins (Excluding Equality Joins)** | **Discrepancies** | **Equality Joins** |
| --- | --- | --- | --- | --- | --- | --- |
| 1 | 385 | 185 | 82 | 33 | 298 | 40 |
| 2 | 264 | 264 | 0 | 0 | 264 | 0 |
| 3 | 30 | 55 | 21 | 0 | 9 | 21 |
| 4 | 77 | 76 | 73 | 0 | 4 | 73 |
| 5 | 615 | 506 | 422 | 404 | 193 | 18 |

**TABLES22. Finding discrepancies-results for some of the tables in the dataset(τ=0.7).**

| **N** | **Threshold** | **TP** | **TN** | **FP** | **FN** | **Accuracy** | **Precision** | **Recall** | **F1 Score** |
| --- | --- | --- | --- | --- | --- | --- | --- | --- | --- |
| 241 | 0.7 | 31 | 203 | 3 | 3 | 0.975 | 0.9118 | 0.9118 | 0.912 |
| 241 | 0.8 | 27 | 204 | 2 | 7 | 0.963 | 0.931 | 0.794 | 0.857 |
| 241 | 0.9 | 23 | 205 | 1 | 11 | 0.950 | 0.958 | 0.676 | 0.793 |

**Table S23. Evaluation Metrics for Identifying Column Relationships.**

| **A_column** | **B_column** | **A_value_orig** | **best_B_value** | **Cosine Similarity** |
| --- | --- | --- | --- | --- |
| title | movie | Crank: High Voltage | Crank 2: High Voltage | 0.818 |
| title | movie | Marley and Me | Marley & Me | 0.978 |
| title | movie | G.I. Joe: The Rise of Cobra | G.I. Joe: Rise of the Cobra | 0.929 |
| title | movie | Julie & Julia | Julie and Julia | 0.947 |
| title | movie | Piranha 3-D | Piranha 3D | 0.977 |
| title | movie | 10,000 B.C. | 10,000 BC | 0.958 |
| title | movie | Fast and Furious | Fast & Furious | 0.987 |
| title | movie | J. Edgar | J. Edgar | 0.902 |
| title | movie | Harold and Kumar Escape from Guantanamo Bay | Harold and Kumar: Escape from Guantanamo Bay | 0.877 |
| title | movie | Dr. Seuss' Horton Hears a Who! | Horton Hears a Who | 0.785 |
| title | movie | The Mummy: Tomb of the Dragon Emperor | The Mummy: Tomb of Dragon Emperor | 0.985 |
| title | movie | Mars Needs Moms | Mars Needs Moms! | 0.967 |
| title | movie | Jackass 3-D | Jackass 3D | 0.986 |
| title | movie | Love & Other Drugs | Love and Other Drugs | 0.986 |
| title | movie | Cats & Dogs: The Revenge of Kitty Galore | Cats and Dogs: The Revenge of Kitty Galore | 0.995 |
| title | movie | Paul Blart: Mall Cop | Paul Blart: Mail Cop | 0.747 |
| title | movie | Fired Up! | Fired Up | 0.885 |
| title | movie | Happy Feet 2 | Happy Feet Two | 0.824 |
| title | movie | Welcome Home, Roscoe Jenkins | Welcome Home Roscoe Jenkins | 0.969 |
| title | movie | Whip It | Whip It! | 0.932 |

**TABLES24. Examples of semantically similar column values between title and movie columns.**

| **A_column** | **B_column** | **A_value_orig** | **best_B_value** | **Cosine Similarity** |
| --- | --- | --- | --- | --- |
| title | movie | I Am Legend | I Am Number Four | 0.255 |
| title | movie | The Bucket List | The Bounty Hunter | 0.291 |
| title | movie | Halloween | Halloween II | 0.506 |
| title | movie | National Treasure: Book of Secrets | Silver Linings Playbook | 0.331 |
| title | movie | Miss Pettigrew Lives for a Day | Miss March | 0.282 |
| title | movie | Hostel: Part II | The Hangover Part II | 0.401 |
| title | movie | Milk | Salt | 0.266 |
| title | movie | Nights in Rodanthe | Date Night | 0.314 |
| title | movie | Pirates of the Caribbean: At World's End | Pirates of the Caribbean: On Stranger Tides | 0.531 |

**TABLES25. Examples of reconciliation discrepancies between title and movie columns.**

| **Method** | **Precision** | **Recall** | **F1-Score** |
| --- | --- | --- | --- |
| Exact Matching | 0.98 | 0.42 | 0.59 |
| Jaccard Similarity | 0.85 | 0.61 | 0.71 |
| Edit Distance | 0.83 | 0.65 | 0.73 |
| TF-IDF / BM25 | 0.81 | 0.70 | 0.75 |
| Sentence-Transformer Baseline | 0.87 | 0.76 | 0.81 |
| DeepJoin-style Column Embeddings | 0.89 | 0.74 | 0.81 |
| Schema-name-only Matching | 0.92 | 0.55 | 0.69 |
| **Proposed Method (Transformer + Metadata)** | 0.91 | 0.83 | 0.87 |

**TABLE S26. Baseline comparison of reconciliation methods.**

| **Metric** | **Annotator 1** | **Annotator 2** | **Annotator 3** |
| --- | --- | --- | --- |
| Label Consistency | 0.84 | 0.83 | 0.82 |
| Semantic Accuracy | 0.87 | 0.85 | 0.86 |
| Domain Knowledge | 0.91 | 0.89 | 0.90 |
| Decision Consistency | 0.82 | 0.81 | 0.83 |
| Confidence | 0.88 | 0.86 | 0.87 |
| Overall Agreement (κ ≈ 0.82) | 0.82 | 0.82 | 0.82 |

**TABLE S27. Evaluation of annotation quality across three independent annotators.**

| **Method** | **Cross-table Reconciliation** | **Semantic Reconciliation** | **Scalable** | **End-to-End Pipeline** |
| --- | --- | --- | --- | --- |
| TaBERT | ✗ | ✓ | ✓ | ✗ |
| SAINT | ✗ | ✓ | ✓ | ✗ |
| TABBIE | ✗ | ✓ | ✓ | ✗ |
| DeepJoin | ✓ | ✓ | ✓ | ✗ |
| WarpGate | ✓ | ✓ | ✓ | ✗ |
| **Proposed Approach** | **✓** | **✓** | **✓** | **✓** |

**TABLE S28. Comparison of the proposed approach with existing methods.**

| **Method** | **Precision** | **Recall** | **F1-Score** |
| --- | --- | --- | --- |
| Exact Matching | 0.97 | 0.42 | 0.58 |
| Jaccard Similarity | 0.95 | 0.50 | 0.65 |
| Edit Distance | 0.93 | 0.48 | 0.63 |
| TF-IDF / BM25 | 0.91 | 0.55 | 0.70 |
| Sentence-Transformers | 0.89 | 0.68 | 0.77 |
| DeepJoin-style Embeddings | 0.88 | 0.66 | 0.75 |
| Schema-name-only Matching | 0.96 | 0.40 | 0.56 |
| **Proposed Method** | **0.95** | **0.80** | **0.87** |

**TABLE S29. Performance comparison against baseline methods.**
